# Humoral immune response to HTLV-1 basic leucine zipper factor (HBZ) in HTLV-1-infected individuals

**DOI:** 10.1186/1742-4690-11-S1-O23

**Published:** 2014-01-07

**Authors:** Yoshimi Enose-Akahata, Anna Abrams, Raya Massoud, Izabela Bialuk, Kory R Johnson, Patrick L Green, Elizabeth M Maloney, Steven Jacobson

**Affiliations:** 1 Viral Immunology Section, Neuroimmunology Branch, National Institute of Neurological Disorders and Stroke, National Institutes of Health, Bethesda, MD, USA; 2 Department of General and Experimental Pathology, Medical University of Bialystok, Bialystok, Poland; 3 Bioinformatics Section, Division of Intramural Research, National Institute of Neurological Disorders and Stroke, National Institutes of Health, Bethesda, MD, USA; 4 Center for Retrovirus Research, The Ohio State University, Columbus, OH, USA; 5 Formerly of the Viral Epidemiology Branch, Division of Cancer Epidemiology and Genetics, National Cancer Institute, National Institutes of Health, Bethesda, MD, USA; 6 Current affiliation: Center for Drug Evaluation and Research, Office of Surveillance and Epidemiology, Food and Drug Administration, Silver Spring, MD, USA

## 

Human T cell lymphotropic virus type I (HTLV-I) infection can lead to development of adult T cell leukemia/lymphoma (ATL) or HTLV-I-associated myelopathy/tropical spastic paraparesis (HAM/TSP) in a subset of infected subjects. HTLV-I basic leucine zipper factor (HBZ) gene has a critical role in HTLV-I infectivity and the development of ATL and HAM/TSP. However, little is known about immune response against HBZ in HTLV-I-infected individuals. In this study, we examined antibody responses against HBZ in serum/plasma samples from 436 subjects including HTLV-I seronegative donors, asymptomatic carriers (AC), ATL, and HAM/TSP patients by the luciferase immunoprecipitation system. The immunoreactivity for HBZ was detected in subsets of all HTLV-I-infected individuals but did not discriminate between AC, ATL and HAM/TSP. However, the frequency of detection of HBZ-specific antibodies in the serum of ATL patients with the chronic subtype was higher than in ATL patients with the lymphomatous subtype. Antibody responses against HBZ were also detected in CSF of HAM/TSP patients with anti-HBZ in serum. Antibody responses against HBZ did not correlate with proviral load and HBZ mRNA expression in HAM/TSP patients, but the presence of HBZ-specific response was associated with reduced CD4^+^ T cell activation in HAM/TSP patients. Moreover, HBZ-specific antibody inhibited lymphoproliferation in PBMC of HAM/TSP patients. This is the first report demonstrating humoral immune response against HBZ associated with HTLV-I infection. Thus, humoral immune response against HBZ might play a role in HTLV-I infection.

